# Study of lead exposure to children residing near a lead–zinc mine

**DOI:** 10.4103/0019-5278.72243

**Published:** 2010-08

**Authors:** Ranjana Choudhari, N. G. Sathwara, V. K. Shivgotra, Shruti Patel, R. A. Rathod, Shagufta Shaikh, M. Idrish Shaikh, Shaswat Dodia, D. J. Parikh, H. N. Saiyed

**Affiliations:** National Institute of Occupational Health, Meghani Nagar, Ahmadabad - 380 016, Gujarat, India

**Keywords:** Blood lead level, lead exposure in children, lead-zinc mine, medical surveillance

## Abstract

This lead exposure study was conducted in a total of 452 school children in the age group of 9–14 years. Two hundred and ninety-eight exposed children came from the villages situated within a 2.5 km radius of the lead–zinc mine whereas the comparative group children were selected from the villages at least 10 km away from mine. Environmental monitoring study suggested that lead levels in air and water samples near the mining areas were within the Central Pollution Control Board prescribed standards. Lead levels in about 80% of the children were less than 10 μg/dl. Medical examination of all children did not show any signs related to lead toxicity but central nervous system-related symptoms, as reported by the subjects during medical examination, were found to be higher in the exposed group when compared with the comparative group. The values of physical growth parameters of the exposed group were comparable with that of the comparative group for both girls and boys. Hence, the physical growth of children was found to be unaffected by the observed level of lead exposure. To safeguard the health of the children residing near the mining area, various preventive and control measures were suggested.

## INTRODUCTION

Lead is ubiquitous in nature. It affects virtually every system in the body. It can damage the nervous system, the renal, and the reproductive systems, cause high blood pressure, and affect growth and development, psychological behavior, and intelligence. Lead exposure in young children is of particular concern because children absorb lead more readily than adults and the developing nervous system of children is particularly vulnerable to the adverse effects of lead. Blood lead levels (Pb-B) as low as 10 μg/dl (microgram/deciliter) are associated with harmful effects on the children’s learning and behavior.[1] Elevated BLL can result in learning disabilities, behavioral problems, and mental retardation.

Lead dust released in the environment during mining and smelting of lead can cause lead exposure to the population living in the vicinity of the mine. Such an open cast mine is situated in Rajasthan State. Hence, a study was undertaken to obtain the base line lead exposure data and its likely health effects on children residing in villages near the mine.

## MATERIALS AND METHODS

This mine is one of the most cost-efficient zinc mines and resource wise, it is estimated that it is the fifth in the world. The mine is an ISO 9001, ISO 14001, and OHSAS 18001 certified unit. The capacity of the mine is 3.75 million MTPA ore production, with 13.54% zinc and 1.97% lead and beneficiation plant to produce zinc and lead concentrates of 53–54% and 60–65%, respectively. To investigate the lead exposure and its health risk/hazards in children due to the mine, medical surveillance and environmental monitoring were carried out as described below:

A total of 452 (exposed – 298 and comparative group – 154) school children in the age group of 9–14 years were randomly selected from different villages. The exposed group comprised of the children staying within a 2.5 km radius of the mine and the comparative group included children from villages situated at least 10 km away from the mine. The study covered medical examination along with lead estimation in the blood sample of each subject. Details of personal and general information along with specific medical examination related to lead toxicity/poisoning were recorded in the pre-designed and tested medical proforma. BLL was used as a biomarker of lead exposure in the study.

Three milliliters of venous blood was collected taking due precaution in a heparinized vacuette at the school premises for lead estimation. The collected blood vacuettes were stored at −4°C in the deep freezer and were transported to the laboratory in dry ice packs. Two milliliters of whole blood was digested in a wet digestion system (Ethios 1600, advanced Microwave lab station made in Italy) using a mixture of 2 ml of nitric acid (ultra pure) and 0.2 ml of hydrogen peroxide while maintaining the time and temperature. The final volume was made to 5 ml using triple distilled water and centrifuged. The clear solution was injected in the atomic absorption spectrophotometer (AAS) to estimate the lead levels in the bloods.

Quality control samples of lead in the blood of different required concentrations were obtained from the Center for Disease Control (CDC), Atlanta, USA. These samples were also run along with the actual analysis of samples to assure the validity of the results.

To assess the environmental exposure levels of lead, representative ambient air and drinking water samples were also collected using standard methods from the villages falling within 2.5 km radius of mine and comparative group of villages which were at least 10 km away from mine and their analysis for lead was also carried out using AAS.

## RESULTS AND DISCUSSION

This will be the first systematic study to know the lead exposure in children residing near the lead producing open cast mine in India.

Environmental monitoring (air and water samples) was carried out of the same villages from where the study subjects were selected. The lead levels in water samples ranged from 6.3 to 13.3 μg/L in different villages situated near the mine, which was less than the prescribed level of 50 μg/L (Central Pollution Control Board [CPCB], 2006).[2] The values of lead in ambient air vary from 0.026 to 1.04 μg/m^3^, which were within the prescribed levels of 1.5μg/m^3^ (CPCB, 2006).[2] These data indicate a lower level of lead exposure due to the mine.

**Figure 1:**
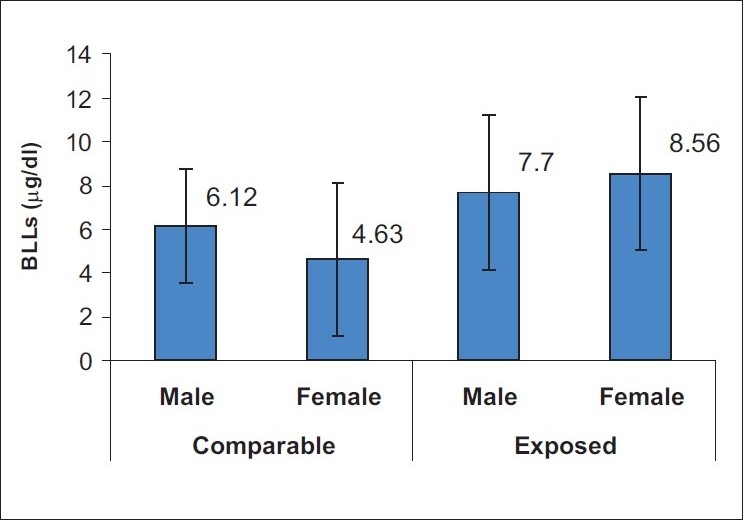
Blood lead levels of children residing in villages near the mine

BLL is an indicator of current exposure and it reflects a dynamic equilibrium between absorption, distribution, and elimination of lead. BLLs in children were used to know the current level of lead exposure. Figure 1 presents the mean BLLs of children residing in villages near the mine. It indicates that the mean BLL in exposed children was 7.7 μg/dl for boys and 8.56 μg/dl for girls while in the comparative group the mean BLLs in boys was 6.12 μg/dl and 4.63 μg/dl in girls. Mean BLLs for the exposed group of children were less than 10 μg/dl, which was considered as a normal value for children as per the CDC,[3] USA. About 80% of the lead values were observed to be less than 10 μg/dl.

Table 1 gives the personal characteristics of the study subjects recorded during the medical examination. This table shows that the exposed group consists of 159 boys and 139 girls of areas located within a 2.5 km radial distance from the mine, whereas 78 boys and 76 girls from areas located at least 10 km away from the mine constitute the comparative group. It clearly demonstrates that both the groups were comparable on various parameters like age, smoking history, tobacco chewing habit, and socioeconomic status. Only three boys from the comparable group (3.8%) and two boys from the exposed group (1.3%) gave history of smoking.

**Table 1:** Personal characteristics of study subjects

Age group (years)	Boys			Girls		
	Comparative	Exposed	Total	Comparative	Exposed	Total
9	Nil	11 (6.9)	11 (4.6)	3 (3.9)	12 (8.6)	15 (6.9)
10	5 (6.4)	20 (12.6)	25 (10.5)	22 (28.9)	25 (18.0)	47 (21.9)
11	20 (25.6)	26 (16.4)	46 (19.4)	14 (18.4)	27 (19.4)	41 (19.1)
12	32 (41.0)	61 (38.4)	93 (39.2)	28 (36.8)	43 (31.0)	71 (33)
13	21 (26.9)	41 (25.8)	62 (26.1)	9 (11.8)	32 (23.0)	38 (19.1)
Total	78	159	237	76	139	215
Smoking habit						
Non-smoker	75 (96.2)	157 (98.7)	232 (97.9)	76 (100)	139 (100)	215 (100)
Smoker	3 (3.8)	2 (1.3)	5 (2.1)	Nil	Nil	Nil
Tobacco chewing						
No	37 (47.4)	109 (68.6)	146 (61.6)	52 (68.4)	129 (92.8)	181(84.2)
Yes	41 (52.6)	50 (30.4)	91 (38.4)	24 (31.6)	10 (7.2)	34 (15.8)
Family income (Rs/month)		Comparative Group			Exposed Group	
<10,000		122 (79.8)			248 (83.2)	
10,000–20,000		30 (19.5)			47 (15.8)	
≥ 20,000		2 (1.3)			3 (1.0)	

Figures in parenthesis indicate percentage

Table 2 presents the prevalence of symptoms in the subjects studied. Gastrointestinal tract, psychological and renal system related symptoms were present respectively in one (0.3%), one (0.3%) and three (1%) of study children from the exposed group but these prevalence were not significant with respect to comparative group. Maximum BLL found in the exposed group was only 14.4 μg/dl. At this BLLs, clinical signs of lead toxicity are not reported, although self-reported central nervous system (CNS)-related symptoms were observed in 22.8% of the exposed children and 13.6% of the comparative subjects, which is statistically significant (*P* < 0.05). Medical examination did not reveal any signs of lead encephalopathy or peripheral neuropathy.

**Table 2:** Prevalence of symptoms in study subjects

Symptoms	Study Population (n=452)							
		C	%	Ex	%	Total	%	Sig.
CNS	Absent	133	86.4	230	77.2	363	80.3	
	Present	21	13.6	68	22.8	89	19.7	
	Total	154	34.1	298	65.9	452	100	0.01998
Renal	Absent	149	96.8	295	99	444	98.2	
	Present	5	3.2	3	1.0	8	1.8	
	Total	154	34.1	298	65.9	452	100	0.08693
Psychological	Absent	154	100	297	99.7	451	99.8	
	Present	..	..	1	0.3	1	0.2	
	Total	154	34.1	298	65.9	452	100	0.47173
GI Tract	Absent	154	100	297	99.7	451	99.8	
	Present	..	..	1	0.3	1	0.2	
	Total	154	34.1	298	65.9	452	100	0.47173

C: Comparative Group, Ex: Exposed Group, %: Percentage

Tables 3 and 4 present the anthropometrics measurements of boys and girls selected in the study. Large cross-sectional studies of young children have shown the association of increased lead concentration with decrease in height, weight, or both.[4,5] The age group-wise evaluation of the somatic growth of the study subjects shows that in both exposed and comparative groups, growth parameters like height and weight were in an increasing trend of values with increasing age, which can be explained as a normal trend. Physical growth parameters of the exposed group were comparable with that of the control group for both girls and boys. The physical growth was not affected at the observed level of lead exposure.

**Table 3:** Age group-wise anthropometrics measurements of boys in the study group

Age group	Number		Age (years)		Height (cm)		Weight (kg)		BMI	
	C	EX	C	EX	C	EX	C	EX	C	EX
9+		11		9.6 ± 0.26		129.7 ± 8.14		25.82 ± 4.05		15.27 ± 0.93
10+	5	20	10.6 ± 0.33	10.5 ± 0.26	132.6 ± 7.71	135.5 ± 8.49	26.00 ± 4.36	26.90 ± 4.31	14.7 ± 1.18	14.57 ± 1.04
11+	20	26	11.72 ± 0.17	11.54 ± 0.31	134.4± 6.79	137.8 ± 6.94	26.70 ± 3.97	28.10 ± 3.84	14.7 ±1.03	14.7 ± 0.91
12+	32	61	12.61 ± 0.25	12.58 ± 0.03	143.4± 9.84	144.1 ± 8.66	30.91 ± 6.26	32.20 ± 5.54	14.87 ± 1.24	15.40 ± 1.25
13+	21	40	13.46 ± 0.28	13.43 ± 0.23	145.4± 7.48	146.1 ± 7.71	31.67 ± 4.48	33.18 ± 6.11	14.90 ±1.13	15.46 ± 1.85

C, comparative group; EX, exposed group.

**Table 4:** Age group-wise anthropometrics measurements of girls in the study group

Age group	Number		Age (years)		Height (cm)		Weight (kg)		BMI	
	C	EX	C	EX	C	EX	C	EX	C	EX
9+	3	12	9.9 ± 0.07	9.43 ± 0.15	134.4 ± 1.53	131.6 ± 8.87	26.70 ± 1.00	23.6± 3.09	14.8± 0.82	13.6± 0.99
10+	22	25	10.55 ± 0.25	10.60 ± 0.20	135.2 ± 7.89	136.46 ± 6.82	27.25 ± 4.80	27.7± 3.74	14.9± 1.51	14.8 ± 1.09
11+	14	27	11.6 ± 0.28	11.6 ± 0.21	138.6 ± 9.23	140.7 ± 7.20	28.1 ± 4.89	30.0 ±5.31	14.5 ± 1.08	15.0± 1.59
12+	28	43	12.6 ± 0.26	12.5 ± 0.26	142.4 ± 8.61	144.0 ± 7.37	31.6 ± 6.99	32.4 ± 6.45	15.4 ± 2.06	15.5 ± 2.11
13+	9	31	13.6 ± 0.25	13.5 ± 0.25	147.3± 9.80	147.3± 9.80	37.8 ± 8.90	35.6 ± 6.06	17.2 ± 2.43	16.1 ± 2.07

C, comparative group; EX, exposed group.

The results of the present study represent preliminary efforts in generating a database of information on lead exposure to children residing near the mine and also to make an attempt to find out the extent and magnitude of health risk consequent to such exposure, considering the different parameters studied.

The main positive finding seen was the CNS-related symptoms as reported by the subjects during the medical examination, which was found to be higher in the exposed group compared with the comparative groups. The difference in both groups was observed to be statistically significant; however, symptoms like headache and giddiness were non-specific in nature and cannot be attributed to only lead exposure. The present BLLs observed in the exposed group of children were not that high, which can give rise to classical signs and symptoms of lead toxicity, like lead line on gums, abdominal pain (lead colic), neurological deficit, joint pain, severe vomiting, anemia, etc. in children. It is reported that the effects of lead on the CNS are embedded in a complex process involving biologic, environmental, familial, and socioeconomic factors. Epidemiological studies cannot, by themselves, establish a causal relationship. Causality is not subject to empirical proof, whether in field or in the laboratory.[6] In India, lead levels and its health effect in children residing in rural areas are not studied much; hence, it is difficult to compare these data with other such data. Bellinger *et al*.[7] reported BLLs 11.1 μg/dl with the range of 2.5–38.3 μg/dl in a cross-sectional study of 74 (4–14 years) children residing in Chennai. Jain and Hu[8] found BLLs between 5 and 20 μg/dl in a retrospective cross-sectional analysis of data from the Indian National Family Health Survey, a population-based study conducted in 1998–1999 in 1081 children (below 3 years) in Mumbai and Delhi. Ahamed *et al*.[9] also reported a mean BLL of 7.47 μg/dl, with the range 2.78–15.0 μg/dl in 62 children (4–12 of age) in Lucknow and nearby areas. Our data are more or less comparable with these data, which were mainly collected from urban cities of India.

Children residing near the mine having poor socio-economic status are considered as a high-risk group to the adverse effect of lead exposure. An additional risk factor is paraoccupational lead exposure to children, which can occur because of their parents working in the lead producing mine. It is reported that BLLs as low as 10 μg/dl are associated with harmful effects on children’s learning and behavior.[1] A number of reviews on the effects of low-level lead exposure on the neuropsychological function of children had been published.[10–13] The outcome of major focus in these reviews was degradation of psychometric intelligence and its relation with low levels of lead exposure.[14] Needle man, in his review article, had given the overall significance of lead poisoning at lower doses of lead that causes no symptoms, particularly in children and fetuses.[15] Nevis has reported a very strong association between pre-school blood lead and subsequent crime rate trend over several decades in the USA, Britain, Canada, France, Australia, Finland, Italy, West Germany, and New Zealand.[16]

Low levels of lead for a longer duration in these children may lead to health risk in the future. Hence, preventive measures and intervention strategies are required to control low-level lead exposure in order to safeguard the children.

The following different suggestions/recommendations were given:

1. Engineering control at the source of the dust exposure should be made powerful so that emission levels of lead dust will remain controlled.
2. Periodic medical examination, including biological monitoring of the children residing near the mine should be carried out.
3. Periodic assessment of community environment (like air, water and soil) near the mine should be carried out.
4. Health education and awareness programs related to lead exposure and health effects should be organized for the villagers residing near the mine.

To safeguard the children from health risk due to lead exposure, the mine management was requested to follow the above suggestions/recommendations and create/maintain the environment (community as well as work environment) clean and safe in the mine. This will help in sustaining the clean environment inside and outside the mining areas.
